# Protective Effect of Bicyclol on Anti-Tuberculosis Drug Induced Liver Injury in Rats

**DOI:** 10.3390/molecules22040524

**Published:** 2017-04-07

**Authors:** Xin Liu, Manman Zhao, Jiaqi Mi, Hui Chen, Li Sheng, Yan Li

**Affiliations:** State Key Laboratory of Active Substances Discovery and Drug Ability Evaluation, State Key Laboratory of Bioactive Substance and Function of Natural Medicines, Beijing Key Laboratory of Non-Clinical Drug Metabolism and PK/PD Study, Department of Drug Metabolism, Institute of Materia Medica, Chinese Academy of Medical Sciences & Peking Union Medical College, Beijing 100050, China; twinsyuanxin@126.com (X.L.); zhaomanman@imm.ac.cn (M.Z.); sylviami@imm.ac.cn (J.M.); chenhui@imm.ac.cn (H.C.)

**Keywords:** bicyclol, isoniazid, rifampicin, pyrazinamide, oxidative stress, CYP2E1, cytokines, mitochondrial dysfunction, HGF

## Abstract

The present study was performed to investigate the effect of bicyclol, a synthetic anti-hepatitis drug with anti-oxidative and anti-inflammatory properties, on anti-tuberculosis (anti-TB) drug-induced liver injury and related mechanisms in rats. Bicyclol was given to rats by gavage 2 h before the oral administration of an anti-TB drug once a day for 30 days. Liver injury was evaluated by biochemical and histopathological examinations. Lipid peroxidation, mitochondrial function, and the activity of antioxidants were measured by spectrophotometric methods. Cytokines expression and CYP2E1 activity were determined by ELISA assay and liquid chromatography–tandem mass spectrometry (LC–MS/MS) analysis. The expressions of hepatic CYP2E1 and hepatocyte growth factor (HGF) were assessed by Western blotting. As a result, bicyclol significantly protected against anti-TB drug-induced liver injury by reducing the elevated serum aminotransferases levels and accumulation of hepatic lipids. Meanwhile, the histopathological changes were also attenuated in rats. The protective effect of bicyclol on anti-TB drug-induced hepatotoxicity was mainly due to its ability to attenuate oxidative stress, suppress the inflammatory cytokines and CYP2E1 expression, up-regulate the expression of HGF, and improve mitochondrial function. Furthermore, administration of bicyclol had no significant effect on the plasma pharmacokinetics of the anti-TB drug in rats.

## 1. Introduction

Tuberculosis (TB), an airborne infectious disease, is one of the major public health issues in the world, especially in developing countries. Approximately two million people die of TB and more than 10 million of new active cases are diagnosed every year [1]. Isoniazid (INH), rifampicin (RIF), and pyrazinamide (PZA) are first-line drugs for anti-TB chemotherapy. However, the hepatotoxicity caused by these drugs is a major concern for clinical treatment due to the long therapy duration and concurrent use of several medications [2].

The mechanism of liver damage induced by the regimen comprised of INH, RIF, and PZA was partially characterized. Toxic intermediaries and reactive oxygen species (ROS) derived from INH biotransformation have been implicated in the progression of hepatotoxicity [3,4]. INH is known to be directly or indirectly metabolized to the toxic metabolites (acetylhydrazine and hydrazine) by *N*-acetyltransferase and amidohydrolase in the liver [5,6,7]. The reactive acetylating species generated by acetylhydrazine are capable of binding covalently with hepatic proteins [8]. Additionally, the hepatotoxicity induced by hydrazine has also been attributed to oxidative stress due to the formation of reactive oxygen species (ROS) mediated by cytochrome P450 2E1 (CYP2E1). CYP2E1, a CYP isoform involved in the production of radicals, has been confirmed to be highly correlated with isoniazid induced hepatotoxicity in both rats and human [9,10]. RIF, as a potent inducer of CYP2E1, could aggravate INH-induced hepatotoxicity by increasing the production of toxic metabolites such as hydrazine, which results in a synergistic effect of INH-induced liver damage [11,12]. PZA is mainly metabolized by liver microsomal amidase and xanthine oxidase, and pyrazinoic acid (PA) and 5-OH-PA, the major toxic metabolites of PZA, are characterized as responsible for PZA-induced hepatotoxicity [13,14].

Growth factors, which are released as a response to injury, play an important role in the repair process of liver after an insult. Hepatocyte growth factor (HGF) represents the first line of defense against hepatotoxicity by regulating the development, survival, and proliferation of hepatocytes through its c-Met receptor. Previous studies showed that HGF/c-Met modulated the activation of critical toxicological transcription factors such as nuclear factor κB (NF-κB) and protein kinase C delta (PKCδ), which can drive the expression of antioxidant and survival genes [15,16], leading to a cell survival response under oxidative stress injuries, such as alcohol metabolism [17].

**Figure 1:**
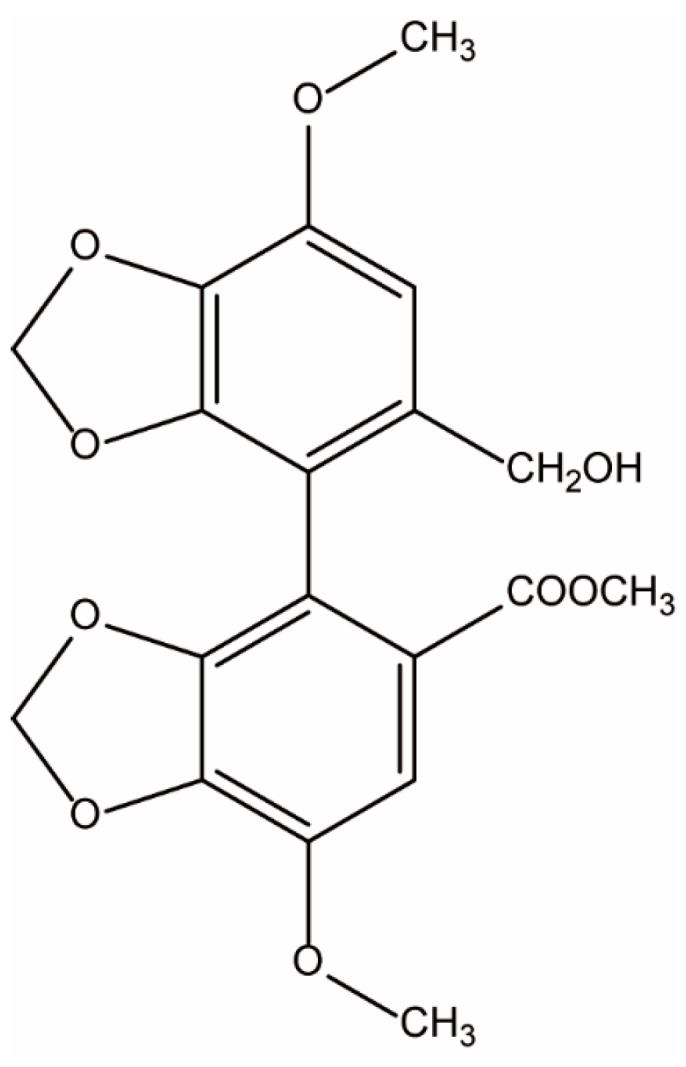
Chemical structure of bicyclol.

Bicyclol (4,4′-dimethoxy-5,6,5′,6′-bis(dimethylene-dioxy)-2-hydroxymethyl-2′-methoxycarbonylbiphenyl; Figure 1) is a synthetic anti-hepatitis drug used for the therapy of chronic hepatitis in China. A previous study indicated that bicyclol had protective effects against experimental liver injury induced by several chemical toxins including CCl_4_, d-galactosamine, concanavalin A, tetracycline, acetaminophen, and alcohol. The hepatoprotective mechanism of bicyclol was related to the clearance of reactive oxygen species (ROS), the regulation of cytokine secretion, the inhibition of apoptosis induced by immunological injury, modulating the disturbance of PPARα pathway, and ameliorating mitochondrial function [18,19,20].

Recent clinical data revealed that bicyclol had been used in the treatment of liver injuries induced by an anti-TB drug, and the damaged liver function in patients was improved by bicyclol administration [21]. However, the mechanisms of the protective effect of bicyclol against liver injury induced by the anti-TB drug are not clear at present. The purpose of this study was to investigate the effect of bicyclol on anti-TB drug-induced liver injury in rats and the related mechanisms. 

## 2. Results

### 2.1. Effect of Bicyclol on Liver Injury Induced by Anti-TB Drug

**Figure 2:**
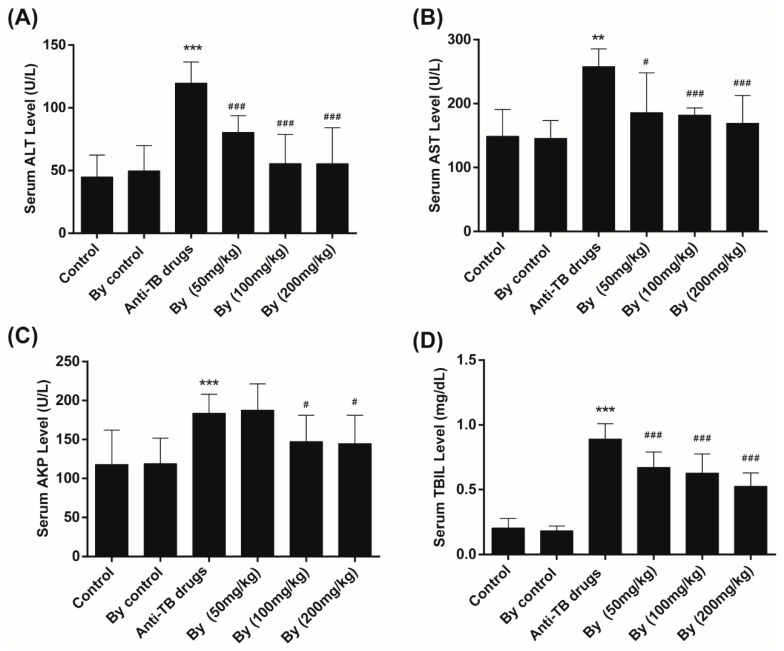
Effect of bicyclol on the elevation of serum ALT (**A**), AST (**B**), AKP (**C**), and TBIL (**D**) in anti-TB drug intoxicated rats. Bicyclol (50, 100, 200 mg/kg) was administered orally to rats for 30 days. Data were expressed as means ± SD (*n* = 10). **, *p* < 0.01, ***, *p* < 0.001 vs. control group; ^#^, *p* < 0.05, ^###^, *p* < 0.001 vs. anti-TB drug intoxicated group.

**Figure 3:**
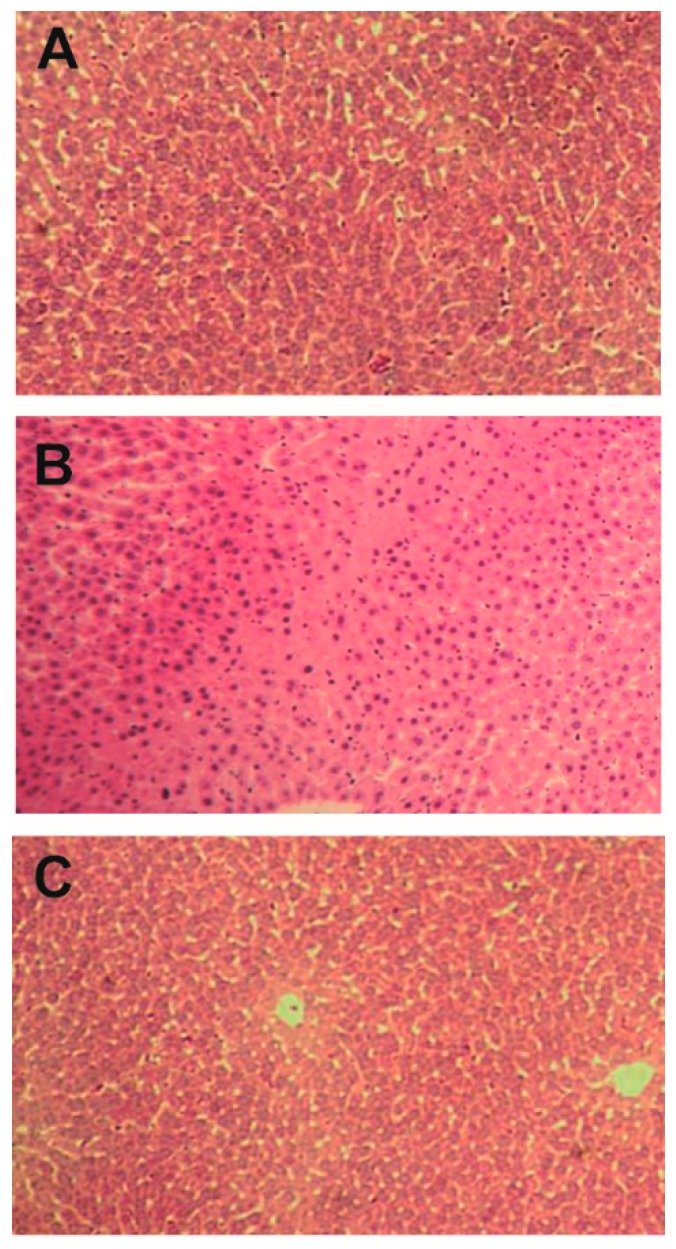
Effect of bicyclol on liver injury induced by anti-TB drug in rats. (**A**) Control rats; (**B**) Anti-TB drug intoxicated rats; and (**C**) Rats treated with bicyclol (200 mg/kg). Hematoxylin and eosin staining; Original magnification, ×40.

Anti-TB drug-induced liver injury was indicated by the elevation of serum alanine aminotransferase (ALT), aspartate aminotransferase (AST), alkaline phosphatase (AKP) and total bilirubin (TBIL) in rats (40.98 ± 14.39 vs. 119.6 ± 16.93, *p <* 0.001; 163.97 ± 46.47 vs. 264.86 ± 28.92, *p* < 0.01; 16.61 ± 2.87 vs. 26.88 ± 4.48, *p <* 0.001 and 0.34 ± 0.17 vs. 0.72 ± 0.08, *p <* 0.001, respectively) and further verified by liver pathological changes characterized by nuclear pleomorphism, increased cellular size, degeneration in hepatocytes and hepatic cords, cytoplasm dissolution, and inflammatory cell infiltration (Figure 2 and Figure 3). Bicyclol (50, 100, 200 mg/kg) significantly reduced the increase of serum ALT, AST, AKP, and TBIL in a dose-dependent manner, while the liver pathological changes were also remarkably improved in anti-TB drug intoxicated rats. In the meantime, no alterations on the above biochemical parameters were observed in rats treated by bicyclol alone (200 mg/kg).

### 2.2. Effect of Bicyclol on Oxidative Stress Induced by Anti-TB Drug

The liver malondialdehyde (MDA) level, as a marker of lipid peroxidation, was significantly increased in rats treated with the anti-TB drug alone, while was simultaneously decreased. In addition, the activities of liver antioxidant enzymes including catalase (CAT), superoxide dismutase (SOD), and glutathione peroxidase (GSH-Px), were also reduced by treatment with the anti-TB drug in rats. Bicyclol showed a significant inhibition of the formation of liver MDA and the depletion of liver glutathione (GSH) contents, as well as the reduction of antioxidant enzymes to the certain extents (Table 1).

### 2.3. Effect of Bicyclol on the Levels of Serum and Hepatic TNF-α and IL-1β in Anti-TB Drug-Intoxicated Rats

**Figure 4:**
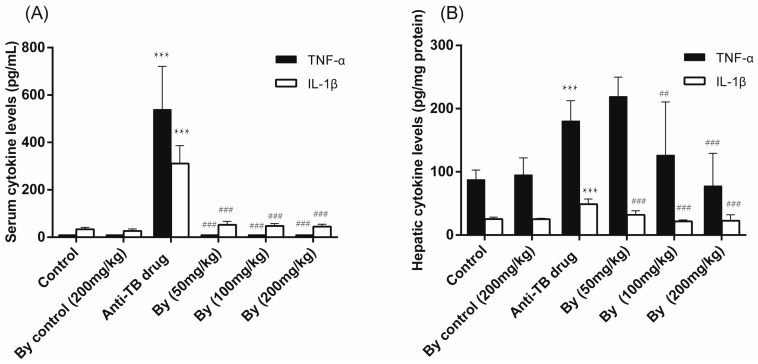
Effects of bicyclol on serum (**A**) and hepatic (**B**) TNF-α and IL-1β protein expression in anti-TB drug intoxicated rats. Bicyclol (50, 100, 200 mg/kg) was administered orally to rats for 30 days. Data were expressed as means ± SD (*n* = 10). ***, *p* < 0.001 vs. control group; ^##^, *p* < 0.01, ^###^, *p* < 0.001 vs. anti-TB drug intoxicated group.

The levels of TNF-α and IL-1β in both serum and the liver were significantly elevated after long-term treatment with the anti-TB drug in rats. Bicyclol alleviated the production of TNF-α and IL-1β in a dose dependent manner (Figure 4).

### 2.4. Effects of Bicyclol on MRC Activity and MPT in Anti-TB Drug-Intoxicated Rats

**Figure 5:**
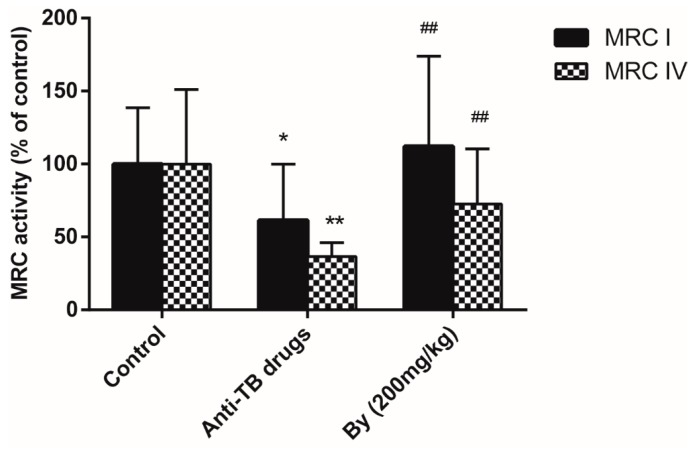
Effect of bicyclol on the activity of liver mitochondrial MRC I and MRC IV in anti-TB drug intoxicated rats. Bicyclol (200 mg/kg) was administered orally to rats for 30 days. Data were expressed as means ± SD (*n* = 10). *, *p* < 0.05, **, *p* < 0.01 vs. control group; ^##^, *p* < 0.01 vs. anti-TB drug intoxicated group.

An increasing number of studies have revealed that mitochondrial dysfunction plays a crucial role in the pathogenesis of anti-TB drug induced liver injury. Thus, mitochondrial respiratory chain (MRC) activity and mitochondrial permeability transition (MPT) were detected in the present study to reflect the mitochondrial function. As a result, a notable reduction in liver MRC complexes I and IV activity (38.6% and 63.4%) was observed after the administration of the anti-TB drug in rats for 30 days. The administration of bicyclol (200 mg/kg) can reverse the decreased activity of hepatic MRC complexes I and IV to the normal levels (Figure 5).

**Figure 6:**
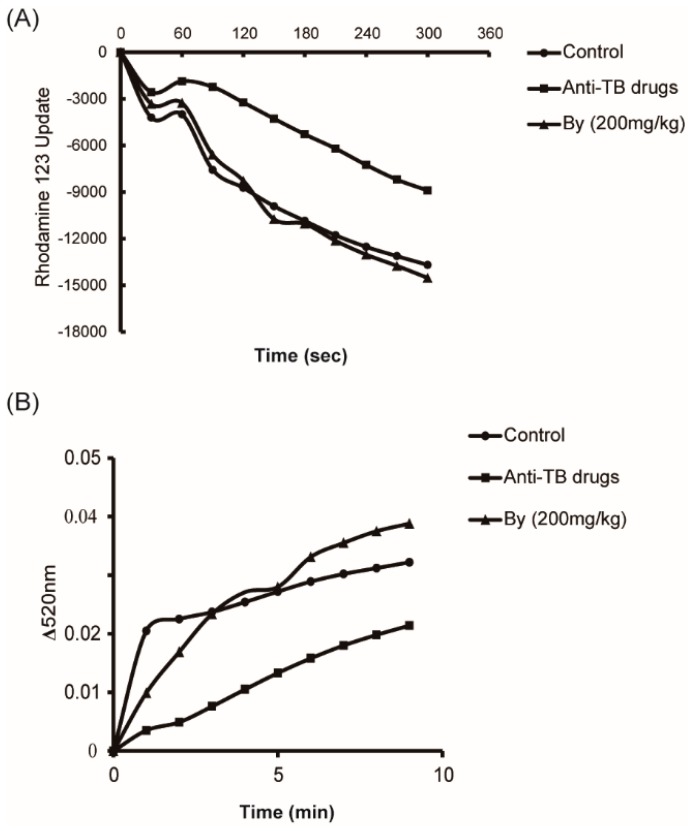
Effect of bicyclol on liver mitochondrial permeability transition (MPT) in anti-TB drug intoxicated rats. Bicyclol (200 mg/kg) was administered orally to rats for 30 days. Mitochondria were isolated immediately after the livers were dissected. (**A**) Mitochondrial membrane potential. (**B**) Mitochondrial swelling.

MPT is an essential indicator for assessing mitochondrial function and the integrity of the mitochondrial membrane. The results showed that the isolated mitochondria had a rapid onset of MPT due to lowered sensitivity to Ca^2+^ in anti-TB drug treated rats. The alteration in MPT was remarkably ameliorated by bicyclol (200 mg/kg) treatment (Figure 6).

### 2.5. Effect of Bicyclol on the Activity and Protein Expression of Hepatic Microsomal CYP2E1 in Anti-TB Drug-Intoxicated Rats

**Figure 7:**
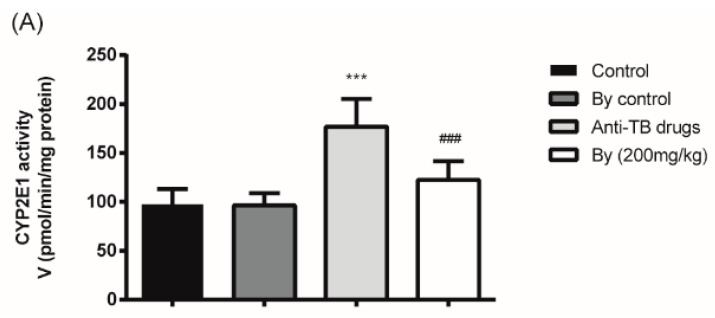

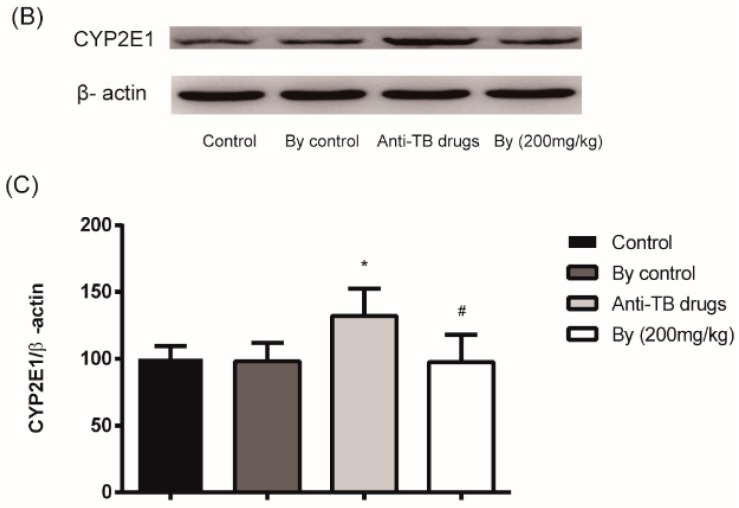
Effect of bicyclol (200 mg/kg) on the activity (A) and protein expression (B and C) of CYP2E1 in anti-TB drug intoxicated rats. *, *p* < 0.05, ***, *p* < 0.001 vs. control group; ^#^, *p* < 0.05, ^###^, *p* < 0.001 vs. anti-TB drug intoxicated group.

As CYP2E1 has been reported to be related to anti-TB drug-induced hepatotoxicity, the activity of hepatic CYP2E1 characterized by the hydroxylation of chlorzoxazone (CZX) was examined. As shown in Figure 7A, the hepatic activity of CYP2E1 in anti-TB drug-intoxicated rats was 1.72 times higher than in the control group (*p* < 0.001). The hepatic microsomal CYP2E1 protein level was also significantly elevated by administration of the anti-TB drug. Co-treatment with bicyclol (200 mg/kg) resulted in a 30.83% decrease in CYP2E1 activity and an inhibition of protein overexpression (Figure 7B,C).

### 2.6. Effect of Bicyclol on the Protein Expression of HGF in Anti-TB Drug-Intoxicated Rats

**Figure 8:**
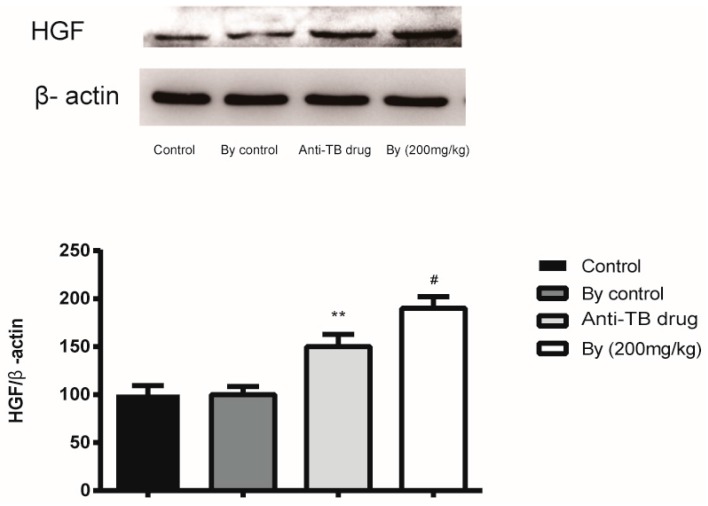
Effect of bicyclol (200 mg/kg) on hepatocyte growth factor (HGF) expression in anti-TB drug intoxicated rats. **, *p* < 0.01 vs. control group; ^#^, *p* < 0.05 vs. anti-TB drug intoxicated group.

As shown in Figure 8, the protein expression of HGF was up-regulated 1.4-fold after anti-TB drug treatment. Co-administration with bicyclol further enhanced the hepatic HGF protein expression, which was about 1.9 times that in the control groups.

### 2.7. Pharmacokinetic Studies

#### 2.7.1. Effects of Bicyclol on the Pharmacokinetics of CZX and 6-OH-CZX in Anti-TB Drug-Intoxicated Rats

**Figure 9:**
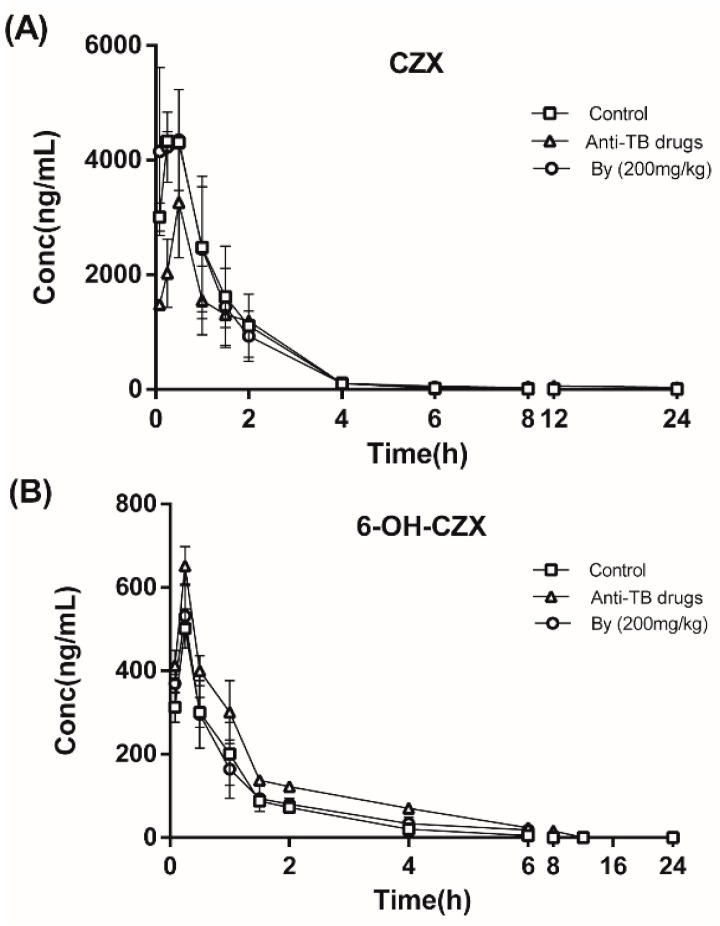
Mean plasma concentration-time curves of CZX (**A**) and 6-OH-CZX (**B**) in the control, anti-TB drug-intoxicated, and bicyclol (200 mg/kg)-treated rats after oral administration of CZX at 20 mg/kg.

The effects of bicyclol on CYP2E1 activity were further investigated by measuring the hydroxylation of CZX to 6-OH-CZX in vivo. The plasma concentration–time curves of CZX and 6-OH-CZX were shown in Figure 9, and the pharmacokinetic parameters were listed in Table 2. Treatment of rats with INH, RIF, and PZA for 30 days resulted in an increase of the area under the plasma concentration–time curve (AUC_0−t_) for 6-OH-CZX and a decrease of AUC_0−t_ for CZX compared with the control group, which indicated that the long-term treatment of rats with anti-TB drug can up-regulate the activity of CYP2E1 and accelerate the hydroxylation of CZX to 6-OH-CZX in rats. Co-administration of bicyclol significantly decreased the AUC_0−t_ of 6-OH-CZX and increased the AUC_0−t_ of CZX to a normal level. These results further confirm that bicyclol can inhibit the elevated CYP2E1 activity induced by treatment with the anti-TB drug in rats.

#### 2.7.2. Effects of Bicyclol on the Pharmacokinetics of INH, RIF, PZA, and PA in Anti-TB Drug-Intoxicated Rats

**Figure 10:**
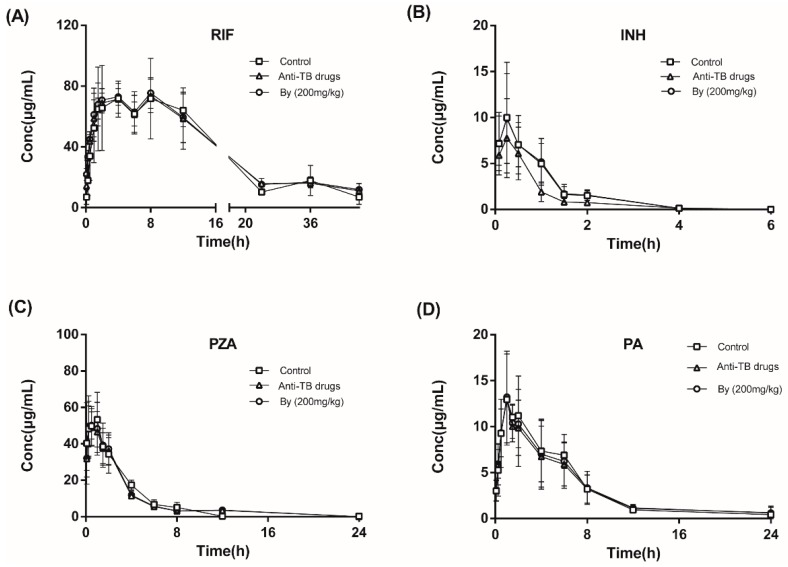
Mean plasma concentration-time curves of RIF (**A**), INH (**B**), PZA (**C**), and PA (**D**) in the control, anti-TB drug-intoxicated, and bicyclol (200 mg/kg) treated rats.

To investigate whether co-treatment with bicyclol could affect the efficacy of anti-TB drug, the effects of bicyclol on the pharmacokinetics of INH, RIF, PZA, and PA in anti-TB drug-intoxicated rats were determined. As shown in Figure 10 and Table 3, co-administration of anti-TB drug to rats for 30 days can decrease the AUC_0−t_ of INH by 29.67%, and the reduction of AUC_0−t_ was significantly prevented by the co-administration of bicyclol. These results indicated that CYP2E1 inhibition affected the metabolism of INH in anti-TB drug-intoxicated rats, and this inhibition can be alleviated by the co-administration of bicyclol. Meanwhile, no significant differences were observed in the plasma pharmacokinetics of RIF, PZA, and PA in any of the tested groups.

## 3. Discussion

Based on the previous studies [11], different times for the animal model of anti-TB drug induced liver injury, ranging from 30 to 90 days, were carried out in the preliminary study. As a result, oral administration of the anti-TB drug to rats for 30 days could steadily induce notable liver injury and no obvious aggravation of hepatic damage was found for longer times. Thereby 30 days was selected for further study.

The results of the present study demonstrated that administration of the anti-TB drug (INH 50 mg/kg, RIF 250 mg/kg, and PZA 100 mg/kg, p.o.) could induce a significant liver injury, as evidenced by the elevation of serum ALT, AST, AKP, and TBIL levels, nuclear pleomorphism, and liver pathological changes. Bicyclol treatment exhibited significant protection against anti-TB drug induced liver damage by attenuating the elevated ALT, AST, AKP, and TBIL levels in a dose-dependent manner, while histopathological changes including nuclear pleomorphism, increased cellular size, degeneration in hepatocytes and hepatic cords, cytoplasm dissolution, and inflammatory cell infiltration were also remarkably improved by bicyclol treatment. In the meantime, no alterations on the above biochemical parameters were observed in rats treated by bicyclol alone (200 mg/kg), which suggested that bicyclol itself had no significant influence on liver function.

The mechanism of hepatotoxicity induced by the anti-TB drug is multifaceted, including oxidative stress to the generation of toxic metabolites and mitochondrial dysfunction [3,4,22].

In our study, the proper balance between oxidant and antioxidant system was found to be disturbed by the anti-TB drug, as indicated by the increased formation of liver MDA and depletion of hepatic GSH and antioxidant enzymes responsible for scavenging hydroperoxides, including SOD, CAT, and GSH-Px. The administration of bicyclol significantly inhibited the increase of hepatic MDA levels, the decrease of tissue GSH levels, and the activities of antioxidant enzyme, which suggested that the imbalance between the oxidant and antioxidant system was reversed by bicyclol to a certain extent. In combination with previous reports concerning bicyclol’s capability to scavenge free radicals generated during different conditions of liver injury [23], it can be confirmed that the attenuation of anti-TB drug-induced oxidative stress by bicyclol was partially due to its ability to restore the balance between the generation and clearance of ROS and strengthen the antioxidant system in rats.

CYP2E1-mediated INH metabolism may result in oxidative stress through the production of toxic metabolites or free radicals and lead to drug-induced hepatotoxicity. Shih et al. reported that CYP2E1 inhibitors, such as kaempferol and disulfiram, had protective effects against anti-TB drug-induced hepatotoxicity through CYP2E1 inhibition. According to our previous unpublished data, pretreatment with bicyclol (300 mg/kg) can decrease mouse liver microsomal CYP2E1 activity induced by alcohol administration and reversed CYP2E1 protein overexpression to approximate a normal level. Thus, we further investigated whether bicyclol exerted its hepatoprotective effect on anti-TB drug-induced liver injury by inhibiting microsomal CYP2E1. As a result, bicyclol suppressed hepatic CYP2E1 at both protein and activity levels in anti-TB drug-intoxicated rats, which is coincident with the further pharmacokinetic study of rats assessed by CZX hydroxylation. In addition, no significant difference between the pharmacokinetics of RIF, PZA, and PA in anti-TB drug-intoxicated rats was observed, compared with normal rats, in the present study, which indicated that co-treatment with bicyclol might not affect the efficacy of the anti-TB drug.

A previous study reported that mitochondrial dysfunction, particularly MRC deficiency, played a key role in the physiopathology of liver injuries. Moreover, the disruption of MRC was considered another crucial source of ROS [20]. The formation of ROS and MRC disturbance formed a vicious circle and accelerated the process of liver injury [24]. Chowdhury et al. [3] also mentioned that oxidative stress in mitochondria and inappropriate MPT are important in the pathogenesis of apoptotic liver cell injury in INH-RIF hepatotoxicity. Our results showed that the activities of mitochondrial oxidative phosphorylation related enzymes (MRC I and MRC IV) were significantly reduced after the administration of the anti-TB drug to rats for 30 days. Furthermore, mitochondrial depolarization and swelling were also observed, which reflected the permeability transition (PT) pores opening in the mitochondrial inner membrane, which was caused by ROS attack [25]. These results supported the view that mitochondrial dysfunction occurred in anti-TB-induced hepatic injury. In our results, bicyclol restored MRC I and IV activity and alleviated mitochondrial swelling and the breakdown of mitochondrial membrane potential. These findings demonstrated that the protective effect of bicyclol against anti-TB drug-induced livery injury might partially be attributed to the improvement of mitochondrial functions.

In addition to oxidative injury, abnormal cytokine metabolism is also a major feature of liver injury [24]. The overexpression of TNF-α and IL-1β was found to be enhanced in both animal models and patients with liver disease [26]. However, there is no evidence as to whether cytokine is involved in the liver injury induced by the anti-TB drug in rats. In this study, the protein levels of TNF-α and IL-1β in both serum and liver tissue were significantly up-regulated by INH/RIF/PZA treatment, and bicyclol significantly attenuated the over expression of TNF-α and IL-1β, which was consistent with the hepatoprotective effect of bicyclol on the liver injury induced by alcohol [19]. In addition, numerous studies have demonstrated that treatment with antioxidants, such as allopurinol, ebselen, and diphenyleneiodonium sulfate can inhibit NF-κB activation and TNF-α expression [27,28]. Thus, we hypothesized that the inhibitory effect of bicyclol on TNF-α expression was at least in part mediated by its antioxidant property.

HGF exerts therapeutic action in a variety of injury and disease models, including acute and chronic renal failure, pulmonary fibrosis, cardiac ischemia, and fibrosis [29]. Likewise, previous studies demonstrated that bicyclol exhibited therapeutic action in various models, including liver fibrosis and renal pathology [30,31]. Combined with our results, the induction of HGF by bicyclol seems to explain, at least in part, the therapeutic effects of bicyclol on anti-TB drug induced liver injury in rats. However, the mechanism of HGF induction by bicyclol remains in further study. 

## 4. Materials and Methods

### 4.1. Reagents

Bicyclol was kindly provided by the Beijing Union Pharmaceutical Plant (purity >99%). RIF and PZA were the products of J&K Scientific Ltd. (Beijing, China). INH, Rhodamine 123, palmitoyl-CoA, oxaloacetate, adenosine diphosphate (ADP), rotenone, antimycin A, ubiquinone-1, chlorzoxazone (CZX), 6-hydroxyl-chlorzoxazone (6-OH-CZX), arachidonic acid, cytochrome c, reduced nicotinamide adenine dinucleotide (NADH), glucose 6-phosphate, β-NADP, glucose 6-phosphate dehydrogenase, phenacetin, resveratrol, and nicotinamide adenine dinucleotide (NAD) were purchased from Sigma Chemical Co. (St Louis, MO, USA). Alanine aminotransferase (ALT) and aspartate aminotransferase (AST) assay kits were obtained from BHKT Chemical Reagent Co., Ltd. (Beijing, China). TBIL, MDA, AKP, SOD, GSH, CAT and GSH-Px kits were purchased from Nanjing Jiancheng Bioengineering Institute (Nanjing, China). Enzyme-linked immunosorbent assay (ELISA) kits were products from eBioscience (San Diego, CA, USA). Other chemicals were of analytical grade and were obtained from the local market. 

### 4.2. Treatment of Animals

Male Wistar rats weighing 180–200 g were obtained from the Beijing Vital River Experimental Animal Co., Ltd. (Beijing, China). The animals were maintained at 22 °C with a 12-h light/dark cycle and had free access to rodent chow and tap water. The research was carried out in accordance with the institutional guidelines and ethics and approved by the Laboratories Institutional Animal Care and Use Committee of the Chinese Academy of Medical Sciences and Peking Union Medical College.

Rats in the bicyclol treated groups were given bicyclol (50, 100, 200 mg/kg, suspended in 0.5% carboxymethyl cellulose) by gavage once a day for 30 days, while other animals received an equal volume of vehicle. All animals were orally administered with the anti-TB drug (INH, 100 mg/kg; RIF, 250 mg/kg; PZA, 50 mg/kg; suspended in 0.5% carboxymethyl cellulose) 2 h after treatment with bicyclol for 30 days, except the rats in the control and bicyclol control groups. The animals were sacrificed by decapitation after 12 h of food deprivation at the end of experiment. Blood samples were collected for the measurement of serum ALT, AST, AKP, and TBIL levels. Liver tissue was rapidly dissected and then cut and fixed in formaldehyde saline (10%) solution for the histological analysis. Another piece of fresh liver tissue was retained for the preparations of mitochondria and microsome. The rest of the liver tissues were snap frozen in liquid nitrogen and then stored at −80 °C until use.

### 4.3. Biochemical Assays

The levels of serum ALT, AST, AKP, and TBIL, the contents of liver MDA and GSH, and the activities of hepatic SOD, CAT, and GSH-PX were measured colorimetrically by commercial kits according to the manufacturer’s instructions.

### 4.4. Liver Histopathological Study

Liver tissues were fixed in formaldehyde saline (10%) solution and embedded in paraplast. Tissue sections (5 μm) were cut and stained by hematoxylin and eosin.

### 4.5. Serum and Hepatic TNF-α and IL-1β Assays

Liver samples for cytokine quantification were prepared by disintegrating the liver tissues in four volumes of ice-cold Ripa buffer (150 mM NaCl, 5 mM EDTA, 50 mM Tris pH 7.4), which contained protease inhibitors (1 μg/mL aprotinin, 10 μg/mL leupeptin, and 1 μg/mL pepstaitn), DNase (0.05 mg/mL), and detergents (0.3% Triton X-100, 0.03% sodium dodecyl sulfate, 0.3% sodium deoxycholate). After being incubated on ice for 30 min, the mixtures were centrifuged twice at 20,000× *g* for 15 min at 4 °C. The supernatants were harvested and then stored at −80 °C until determination. Liver lysates were adjusted to equal protein concentrations after quantification by Bradford assay. Serum and hepatic cytokines were detected by ELISA kits, and the results were expressed as pg/mL and pg/mg protein, respectively.

### 4.6. Preparation of Hepatic Subcellular Fractions

After treatment of the rats for 30 days, rat liver microsomes (RLMs) and mitochondria were prepared as described previously [20,32]. Microsomal and mitochondrial samples were finally aliquoted and frozen at −80 °C until use. The protein concentration was determined by Bradford assay, and the content of CYP450 in liver microsomes was then determined by a spectrophotometric method [33].

### 4.7. Mitochondrial Permeability Transition (MPT) Assay

The mitochondrial membrane potential experiment was carried out using Rhodamine 123 as a fluorescent probe [34]. After the detection of the basic fluorescent intensity of the reaction mixture (15 mM sucrose, 5 mM succinate, 5 mM MgCl_2_, 5 mM K_2_HPO_4_, 20 mM HEPES, and 0.5 mM Rhodamine 123) at 503/527 nm, mitochondria were added at a final concentration of 0.1 mg/mL protein. After incubation for 30 s, the alteration of fluorescent intensity was monitored for 5 min.

Mitochondrial swelling was determined according to the method of Hunter et al. [35]. The reaction medium (0.5 mg/mL liver mitochondrial protein, 250 mM sucrose, 5 mM KH_2_PO_4_, and 3 mM succinate) was pre-incubated at 25 °C for 2 min. and then the absorbance at 520 nm was immediately monitored on a spectrophotometer for 10 min after adding 300 mM CaCl_2_.

### 4.8. Measurement of Enzyme Activities

Hepatic CYP2E1 activity was determined by measuring the hydroxylation of CZX to 6-OH-CZX, as described previously by Kharasch et al. [36]. After a 5 min preincubation step, the reactions were started by adding a NADPH-generating system (10 mM glucose 6-phosphate, 1 mM NADP^+^, and 1 unit of glucose 6-phosphate dehydrogenase) and quenched by twice the volume of ice-cold acetonitrile, containing internal standard (IS), after incubating at 37 °C for 10 min. All samples were tested in triplicate. The incubation mixtures were centrifuged at 14,000 *g* for 5 min, and 5 μL of the aliquots were then analyzed by liquid chromatography–tandem mass spectrometry (LC–MS/MS).

The activity of NADH-ubiquinone reductase (MRC I) and cytochrome c oxidase (MRC IV) was detected according to Przedborski et al. [37].

### 4.9. Western Blotting Analysis

The extracts of liver tissue were prepared by homogenization in lysis buffer (50 mM Tris, pH 7.5, 150 mM sodium chloride, 1 mM phenylmethylsulfonyl fluoride, 1 mM sodium orthovanadate, 1% Nonidet P-40, 50 mM sodium fluoride, 10 μg/mL proteinase inhibitors mixture, 10% glycerol) at 4 °C and then centrifugated at 20,000 *g* at 4 °C for 10 min. After the quantification of protein concentrations, the supernatants were mixed with Laemmli loading buffer, boiled for 4 min, and then subjected to Western blot analysis. The membranes were blotted against the primary antibodies at 4 °C for 16 h, washed with 0.1% (*v/v*) Tween-20 in Tris-buffered saline (pH 7.4), and incubated with horseradish peroxidase-conjugated secondary antibodies for 45 min. Protein bands were visualized by the enhanced chemiluminescence reaction method (Applygen Technologies Inc., Beijing, China). The densities of the bands were measured by the GelPro32 software program (Media Cybernetics, Marlow, UK). Western blotting analysis was carried out at least three times for the protein of interest.

### 4.10. Pharmacokinetic Studies

#### 4.10.1. Pharmacokinetics of CZX and 6-OH-CZX in Rats

After treatment for 30 days, rats in the control, anti-TB drug intoxicated, and bicyclol treatment (200 mg/kg) groups were given CZX orally at a dose of 20 mg/kg. Blood samples were collected in heparinized microcentrifuge tubes at intervals of 0.08, 0.25, 0.5, 1, 1.5, 2, 4, 6, 8, 12, and 24 h after the administration of CZX. Plasma samples were immediately obtained by centrifugation at 5000 rpm for 10 min and then stored at −80 °C until further analysis. 

#### 4.10.2. Pharmacokinetics of INH, RIF, PZA and PA in Rats

Rats in the groups of control, anti-TB drug intoxicated, and bicyclol treatment (200 mg/kg) were administered INH/RIF/PZA orally at a dose of 50/250/100 mg/kg after treatment for 30 days. Approximately 0.2 mL of blood samples were collected in heparinized 1.5 mL polythene tubes by orbital bleeding via capillary tubes at intervals of 0.08, 0.25, 0.5, 1, 1.5, 2, 4, 6, 8, 12, 24, 36, and 48 h after dosing. Plasma was immediately obtained by centrifugation at 5000 rpm for 10 min and stored as mentioned above.

#### 4.10.3. LC–MS/MS Analysis

A 20 μL of IS working solution (resveratrol, 500 ng/mL) and 180 μL acetonitrile were mixed with 100 μL of rat plasma or the incubation mixture of rat liver microsomes. After centrifugation at 14,000 rpm for 5 min, an aliquot of 5 μL was injected into the HPLC–MS/MS system for the analysis of CZX and 6-OH-CZX.

The HPLC–MS/MS system consists of a Surveyor Autosampler, a Surveyor LC pump, a TSQ Quantum AccesseTM triple quadrupole mass spectrometer with an electrospray ionization (ESI) source, and Xcalibur 1.4 software for data acquisition and analysis (Thermo Finigan, San Jose, CA, USA). The analytical column used was a ZorbaxSB-C18 column (3.5 μm, 2.1 mm × 100 mm, Agilent, Santa Clara, CA, USA). The separation was performed on a ZorbaxSB-C18 column (3.5 μm, 2.1 mm × 100 mm) with a gradient elution of acetonitrile/water at a flow rate of 0.2 mL/min. The product ions were recorded using a negative ion detection mode. The monitored ions and collision energies were *m*/*z* 184.0–120.0 and 18 eV for 6-OH-CZX, *m*/*z* 167.9–131.9, and 21 eV for CZX, and 227–143.6 and 29 eV for resveratrol (IS). The assay was linear up to 5000 ng/mL for CZX and 1000 ng/mL for 6-OH-CZX, with the lowest limit of quantification (LLOQ) being 10 ng/mL (1/X weighting).

For the analysis of INH, RIF, PZA, and PA in rat plasma, the samples were pretreated similarly to the method described above. The mobile phase consisted of solvent A (0.1% formic acid in methanol) and solvent B (0.1% formic acid in water). A gradient elution was applied by setting solvent A at 30% in 0.5 min and then increasing it by 95% in the next 2 min, followed by re-equilibration at 30% until 9 min. The flow rate was at 0.2 mL/min with an operating temperature of 30 °C. The monitored ions and collision energies were *m*/*z* 138.1–121.1 and 15 eV for INH, *m*/*z* 823.4–791.5 and 26 eV for RIF, *m*/*z* 124.0–81.1 and 24 eV for PZA, *m*/*z* 124.0–81.1 and 24 eV for PZA, *m*/*z* 180.0–110.0 and 18 eV for phenacetin (IS) in positive ion mode, and *m*/*z* 123.0–79.0 and 16 eV for PA in negative ion mode. The calibration curve was shown to be quadratic up to 100 μg/mL for the LLOQ of 50 ng/mL for RIF and PZA (1/X weighting) and 20 μg/mL for the LLOQ of 50 ng/ mL for INH and PA (1/X weighting).

Low-, mid-, and high-level quality control samples were used for the analytes in HPLC–MS/MS analysis to provide an indication of accuracy in the present study.

### 4.11. Statistical Analysis

All data were expressed as mean ± SD. Between-group comparison was evaluated by one-way ANOVA analysis. A *p* value < 0.05 was considered statistically significant. The pharmacokinetic parameters of CZX, 6-OH-CZX, INH, RIF, PZA, and PA were obtained from noncompartmental analysis using Phoenix WinNonlin software (version 6.3, Certara Corporation, St. Louis, MO, USA). 

## 5. Conclusions

Obvious hepatic injury was observed after the treatment of rats with the anti-TB drug for 30 days. Bicyclol showed a significant protective effect on anti-TB-induced liver injury. The hepatoprotective action of bicyclol might be associated with its ability to attenuate oxidative stress, suppress cytokine overexpression, modulate CYP2E1, and induct HGF. Furthermore, the administration of bicyclol did not affect the plasma pharmacokinetics of RIF, PZA, and PA in rats.

## Figures and Tables

**Table 1:** Influence of bicyclol on liver GSH, MDA contents, and SOD, CAT, and GSH-Px activities in anti-TB drug-intoxicated rats.

Groups	Liver GSH (mg/g Tissue)	Liver MDA (nmol/mg Tissue)	Liver SOD (U/mg Tissue)	Liver CAT (U/mg Tissue)	Liver GSH-Px (U/mg Tissue)
Normal control	12.77 ± 6.71	3.96 ± 1.13	627.72 ± 320.72	48.19 ± 17.33	256.32 ± 43.45
By control	14.54 ± 12.16	3.97 ± 1.01	766.85 ± 945.57	42.52 ± 13.38	269.18 ± 48.94
Anti-TB drug	4.14 ± 1.28 ***	9.03 ± 2.71 ***	301.82 ± 67.17 **	22.43 ± 11.96 ***	182.26 ± 18.28 *
By (50 mg/kg)	7.84 ± 4.94	4.35 ± 0.85 ^###^	493.61 ± 364.85	29.30 ± 7.59	260.21 ± 44.85
By (100 mg/kg)	6.53 ± 2.27 ^#^	4.07 ± 0.69 ^###^	471.62 ± 215.07	31.09 ± 10.41 ^##^	280.08 ± 68.25
By (200 mg/kg)	8.01 ± 2.40 ^##^	3.81 ± 0.84 ^###^	487.15 ± 152.62 ^##^	40.61 ± 9.80 ^###^	427.77 ± 53.28 ^##^

*, *p* < 0.05 vs. normal control group; **, *p* < 0.01 vs. normal control group; ***, *p* < 0.001 vs. normal control group; ^#^, *p* < 0.05; ^##^, *p* < 0.01 vs. anti-TB drug intoxicated group; ^###^, *p* < 0.001 vs. anti-TB drugs intoxicated group.

**Table 2:** Effects of bicyclol on the pharmacokinetics of CZX and 6-OH-CZX in anti-TB drug-intoxicated rats after oral administration of CZX at 20 mg/kg.

Parameters	Unit	CZX			6-OH-CZX		
		Normal Control	Anti-TB Drug	By (200 mg/kg)	Normal Control	Anti-TB Drug	By (200 mg/kg)
*t_1/2z_*	h	2.37 ± 0.83	2.81 ± 0.63	2.24 ± 0.19	-	-	-
*T_max_*	h	0.42 ± 0.14	0.50 ± 0.00	0.48 ± 0.21	0.25 ± 0.00	0.25 ± 0.00	0.25 ± 0.00
C_max_	μg/L	4402.13 ± 108.37	3263.51 ± 965.98	4395.34 ± 537.32	501.44 ± 46.93	651.44 ± 46.93	521.72 ± 76.42
AUC_(0−t)_	μg/L*h	6765.56 ± 1715.18	5180.76 ± 542.58	6670.83 ± 1299.81	551.7 ± 84.89	954.45 ± 68.02	534.81 ± 109.62
V_z_/F	L/kg	10.57 ± 4.57	9.67 ± 1.60	10.51 ± 18.85	-	-	-
CL_z_/F	L/h/kg	3.06 ± 0.70	2.41 ± 0.16	3.04 ± 0.56	-	-	-
MRT_(0−t)_	h	1.58 ± 0.12	1.48 ± 1.13	1.82 ± 0.79	1.35 ± 0.41	2.19 ± 0.04	1.97 ± 0.48

**Table 3:** Pharmacokinetic parameters of isoniazid (INH), rifampicin (RIF), pyrazinamide (PZA), and pyrazinoic acid (PA) in normal, anti-TB drug-intoxicated, and bicyclol (200 mg/kg)-treated rats for 30 days.

Parameters		*t_1/2z_*	*T_max_*	C_max_	AUC_(0−t)_	V_z_	CL_z_	MRT_(0−t)_
Unit		h	h	μg/mL	μg/mL·h	L/kg	L/h/kg	h
RIF	Normal control	6.97 ± 5.11	4.75 ± 4.02	205.72 ± 220.27	1394.39 ± 836.99	2.18 ± 0.41	0.14 ± 0.05	11.02 ± 8.34
	Anti-TB drug	15.31 ± 1.86	4.5 ± 3.28	79.40 ± 10.38	1999.83 ± 246.72	3.26 ± 0.85	0.13 ± 0.01	21.94 ± 2.42
	By (200 mg/kg)	17.29 ± 5.17	4.5 ± 3.28	76.21 ± 2.27	1901.43 ± 140.29	3.16 ± 0.63	0.13 ± 0.02	23.88 ± 5.23
INH	Normal control	0.60 ± 0.08	0.50 ± 0.43	10.90 ± 4.91	11.02 ± 2.78	4.19 ± 1.78	4.76 ± 1.32	0.95 ± 0.19
	Anti-TB drug	0.56 ± 0.21	0.19 ± 0.10	8.25 ± 3.60	7.75 ± 1.74	21.82 ± 29.56	6.68 ± 1.59	1.90 ± 1.92
	By (200 mg/kg)	0.60 ± 0.08	0.50 ± 0.43	10.89 ± 3.53	11.22 ± 2.09	4.00 ± 1.42	4.57 ± 0.95	0.95 ± 0.19
PZA	Normal control	2.93 ± 0.49	0.53 ± 0.46	54.10 ± 14.29	192.02 ± 40.57	2.31 ± 0.79	0.54 ± 0.11	3.23 ± 0.35
	Anti-TB drug	2.28 ± 0.16	0.50 ± 0.43	57.07 ± 12.57	188.24 ± 34.24	1.80 ± 0.37	0.54 ± 0.09	3.70 ± 0.35
	By (200 mg/kg)	2.28 ± 0.16	0.50 ± 0.43	58.21 ± 7.28	194.97 ± 33.3	1.76 ± 0.40	0.53 ± 0.09	3.70 ± 0.35
PA	Normal control	4.78 ± 0.69	1.33 ± 0.58	14.80 ± 1.80	82.28 ± 16.1	8.61 ± 2.20	1.25 ± 0.26	6.15 ± 0.92
	Anti-TB drug	5.03 ± 1.72	1.17 ± 0.29	13.54 ± 3.94	82.40 ± 22.1	9.04 ± 2.74	1.29 ± 0.41	7.42 ± 2.74
	By (200 mg/kg)	5.03 ± 1.72	1.17 ± 0.29	13.88 ± 3.21	84.66 ± 15.97	8.89 ± 2.89	1.23 ± 0.28	7.40 ± 2.74
